# Hypovitaminosis D, oral potentially malignant disorders, and oral squamous cell carcinoma: a systematic review

**DOI:** 10.4317/medoral.25049

**Published:** 2022-02-20

**Authors:** Andrea Maturana-Ramírez, Juan Aitken-Saavedra, Arantxa Ladrón de Guevara-Benítez, Iris Espinoza-Santander

**Affiliations:** 1 Department of Oral Pathology and Medicine, Faculty of Dentistry, Universidad de Chile, Santiago, Chile; 2 Therapeutic Diagnostic Center Odontology and Pathological Anatomy Service, Hospital Complex San Jose, Santiago, Chile; 3 San Camilo Hospital, San Felipe, Chile; 4 Undergraduate, Faculty of Dentistry, Universidad de of Chile, Santiago, Chile

## Abstract

**Background:**

Oral squamous cell carcinoma (OSCC) and potentially malignant oral disorders (OPMDs) could be associated with low levels of vitamin D. This systematic review aimed to determine the relationship between serum levels of vitamin D with OPMDs and OSCC.

**Material and Methods:**

This review was conducted according to Cochrane guidelines (PROSPERO CRD42020207382) on literature retrieved from the PubMed, Cochrane, and Web of Science databases. The antecedents extracted were study design, methodology, sample (country, number of patients, age, and sex), oral manifestations (type of lesion, location, prevalence, and follow-up), serum vitamin D levels or use of vitamin D supplements, results, and conclusions.

**Results:**

Twelve articles were selected. Some of the most relevant findings were alterations in vitamin D could favor the progress of OPMDs to OSCC. Higher levels of vitamin D can increase levels of anti-inflammatory mediators, CD4+ T lymphocytes and CD8+ T lymphocytes and CD3+ T lymphocytes in intratumoral tissue. The normalization of vitamin D levels in patients with OSCC can increased cytotoxic activity of natural killer cells, favoring antitumor immune response. Vitamin D supplemented can lower adverse effects associated with chemotherapy like mucositis and pain. Tobacco can increase risk of developing OSCC altering vitamin D levels.

**Conclusions:**

Hypovitaminosis D could increase risk of developing OSCC from OPMDs, thus altering the immune response and it is associated with a lower survival rate in patients with OSCC, a greater recurrence of tumors in patients who underwent surgical treatment, and an increase in adverse reactions to chemotherapy. The use of vitamin D supplements can be a complement to primary therapy to prevent the recurrence of lesions and reduce adverse events associated with treatment.

** Key words:**Oral squamous cell carcinoma (OSCC), Oral potentially malignant disorders (OPMDs), Vitamin D, Oral cancer.

## Introduction

Cancer is currently the second leading cause of death in the world and is expected to take first place in many countries by 2030 (1). Oral squamous cell carcinoma (OSCC) corresponds to 90% of oral cancers. It features an aggressive pathology, usually with a poor prognosis due to its late diagnosis since the lesions are usually detected at stage III or IV (2), radicalizing the treatment options and negatively impacting the quality of life of patients. Five-year survival Figures for oral cancer have not improved (50–60% overall survival) (3). Oral potentially malignant disorders (OPMDs) are characterized by morphological alterations indicating an increased potential for malignant transformation. They are further associated with a greater risk of developing cancer, i.e., OSCC, at any site of the oral mucosa (4). Thus, although some lesions present a greater risk of progressing to OSCC, the risk is also in apparently healthy mucosa, in fact, may arise at a site distant to an existing premalignant lesion (5,6).

Vitamin D as having different anticancer effects (7). Vitamin D is a steroid hormone that has two main forms in humans, vitamin D3 or cholecalciferol, which is synthesized in the skin after exposure to sunlight or ultraviolet light, and vitamin D2 or ergocalciferol, which is obtained through the irradiation of plants, plant materials, or food (8). Serum vitamin D is metabolized in the liver and then in the kidneys to obtain its active metabolite, 1,25(OH)2D, which binds to vitamin D receptors (VDR) widely distributed in different tissues, promoting cell differentiation, inhibiting the proliferation of carcinogenic cells, and promoting anti-inflammatory, pro-apoptotic, and antiangiogenic effects (9). On the other hand, there have suggested a relationship between hypovitaminosis D with an increased risk of cancer incidence and associated mortality (9). Optimal vitamin D levels have been associated with a lower likelihood of developing OSCC and longer survival after diagnosis (10). Although a possible association between hypovitaminosis D and the potential for malignancy has been suggested, the evidence is scarce and contradictory. The objective of this systematic review was to establish the association between hypovitaminosis D with potentially malignant disorders and OSCC.

## Material and Methods

This systematic review was conducted according to the guidelines of the Cochrane Handbook for Systematic Reviews of Interventions, following the four-phase flow chart of the Preferred Reporting Items for Systematic Reviews and Meta-Analyses Statement and it is registered in the PROSPERO International Prospective Registry of Systematic Reviews under Code Number CRD42020207382. Reviewers conducted the review according to PubMed (National Library of Medicine), Cochrane (Elsevier), and Web of Science (Thomson Reuters) databases. In addition, the reference lists of selected articles were further searched for additional articles. Articles published in English, Spanish, and Portuguese between 2009 and 2020 were considered. The search strategy is described in (Supplement 1).

All studies reported in English, Portuguese, or Spanish that met the inclusion or problem, intervention, comparison, and outcome criteria were analyzed. Clinical trials and clinical, population, case-control, and cohort studies were included. The studies should have been carried out in humans over 18 years of age where measurements of serum vitamin D levels were obtained, or vitamin D supplementation was included in the treatment of patients with a histopathological diagnosis of a potentially malignant disorder or OSCC. Animal studies, *in vitro* or *in vivo* studies, and previous systematic reviews were excluded. When the search results were obtained, the titles and abstracts were assessed to select the articles that showed the greatest agreement, which were subsequently retrieved in full to verify the degree of compliance with the eligibility criteria (inclusion and exclusion criteria). The antecedents extracted from each study were language, study design, methodology and eligibility criteria, sample (country, number of patients, age, and sex), oral manifestations (type of lesion, location, prevalence, and follow-up), vitamin D serum evaluation or use of vitamin D supplements, results, and conclusions. A random-effects model was used for analysis. Due to the high degree of heterogeneity in terms of study design and methodology, performing a quantitative analysis of the meta-analysis was considered inappropriate.

## Results

**Figure 1:**
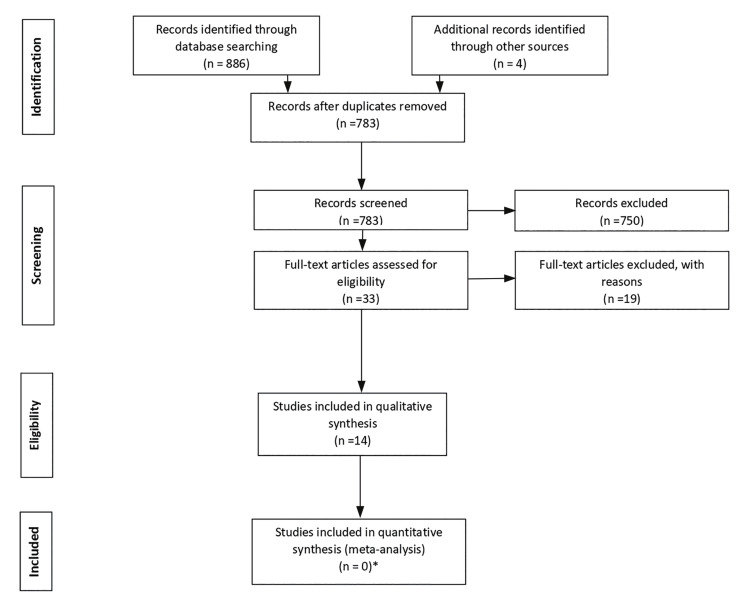
Search flowchart according to the PRISMA Statement.

A total of 886 articles were retrieved through the indicated platforms, and 4 articles were identified through manual methods. Of the 886 articles, 107 duplicates were removed. The remaining 783 articles were filtered by title, abstract, and inclusion and eligibility criteria. After an exhaustive evaluation, 12 texts were finally selected for analysis (11-21). The flow chart for the methodology is shown in Fig. 1.

The analysis of the 12 articles is summarized in Table 1. Four studies were conducted in Asia (11,12,14,17), 4 in Europe (10,13,15,18), 4 in North America (United States) (16,19-21), Six were case-control studies (11-13,15-17), 3 were cohort (10,14,18), and 3 were clinical trials (19-21). The methods of measuring serum vitamin D levels included electrochemiluminescence (n = 1) (12), chemiluminescence (n = 2) (13,14), enzyme-linked immunosorbent assays (n = 1) (17), liquid chromatography (n = 2) (10,18), and radioimmunoassay (n = 1) (17). Of the articles that specified the age of the patients (10,16-18,20,21), the age range was between 20 and 92 years, whereas that for patients with OPMDs was between 37.2 and 60.7 years.

**Table 1:**
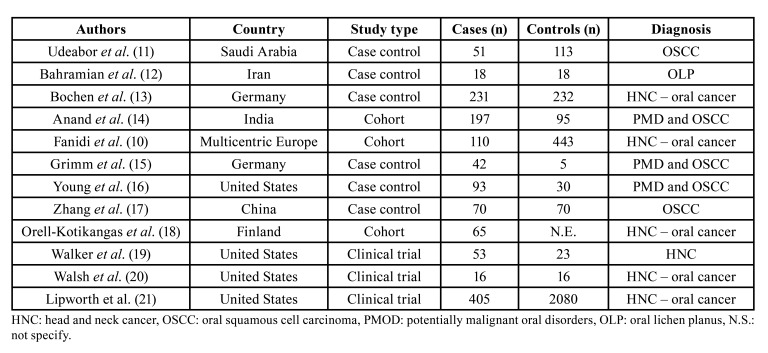
Review’s included articles

In patients with OSCC, the age range was between 48 and 63 years. In the articles that specified the distribution by sex (10-16,18,20,21), male patients were predominant, ranging between 57.1% and 82% of the sample population. In those with a diagnosis of oral lichen planus (OLP) (12), the female sex was predominant, between 61.1% and 70% of the population. Of the articles that specified the lesion site (n = 6) (11,13-15,18,20), for OSCC, the most frequent sites were the floor of the mouth, jugal mucosa, ridge, and tongue. Regarding the habits of the patients, 4 articles listed smoking (10,17,18,21), and of these, 3 also listed alcohol consumption (10,17,21). Three articles associated these habits with serum vitamin D levels (10,18,21), and 1 identified difference in smokers with low vitamin D levels (18). Two articles observed a higher risk of developing cancer in smokers with low vitamin D (10,21).

Of the articles that considered patients with OSCC (n = 11) (10,11,13,14,21), 5 exclusively considered patients with OSCC (11,14,15-17), whereas 6 also included other locals of squamous cell carcinoma (SCC) such as oropharynx, nasopharynx, hypopharynx, and larynx (10,13,18-21). The results of the 5 articles that specified the measurement methods and values used for vitamin D are summarized in Table 2.

Three articles evaluated variations in the immune response of the subjects according to their vitamin D levels (13,16,19). Young *et al*. indicated that patients supplemented weekly with 12 µg of 1,25(OH)2D 3 in a 3-week period had higher levels of anti-inflammatory mediators such as adiponectin and a decrease in pro-inflammatory mediators such as IL-6, IL-17, and leptin (16). Bochen *et al*. and Walsh *et al*. indicated an increase in CD4+ T lymphocytes and CD8+ T lymphocytes in the intratumoral tissue of patients with SCC who received vitamin D supplementation (13,20). Bochen *et al*. evaluated other cell populations, observing an increase in CD3+ T lymphocytes, natural killer cells, macrophages, and M1 macrophages in intratumoral tissue (13).

**Table 2:**
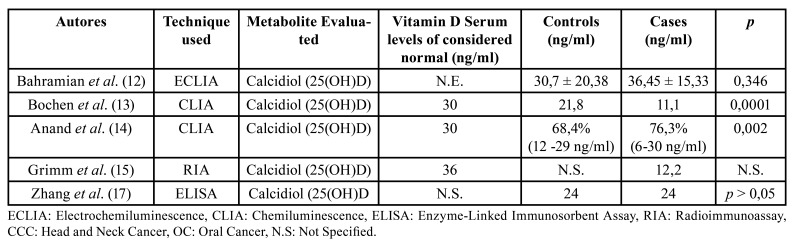
Evaluation of vitamin D levels

Grimm *et al*. evaluated VDR in relation to its expression in oral keratinocytes in case and control patients, showing that patients with OLP had an up to 50% lower expression rate of receptors in the oral mucosa compared with those without lesions (15). Furthermore, Anand *et al*. associated low vitamin D levels with greater severity of post-chemotherapy adverse reactions such as edema, erythema, ulcers, and pain (14). They found that increasing serum vitamin D levels mitigated the adverse effects associated with chemotherapy in patients with OSCC such as oral mucositis and pain and improved swallowing and quality of life.

## Discussion

Our results showed that Asia is one of the continents with the highest number of publications, probably because it has a high incidence of OSCC in the world (22). Studies on patients with OPMDs used different methodologies to histopathologically characterize oral epithelial dysplasias. In 2017, WHO presented two ways of classifying dysplasias: graduation of dysplasia into “mild,” “moderate,” and “severe” categories (WHO dysplasia grade) and a binary system of “low” and “high” grade (4). The use of the latter classification could simplify the comparison between patients and avoid biases. Indeed, the use of different criteria to assess the degree of dysplasia and vitamin D levels makes the comparison of the different articles difficult.

Patients with OPMDs were over 40 years of age (23), and the frequency of OSCC increased between the fifth and sixth decades of life (24,25). OSCC and OPMDs are more frequent in men, except in OLP, which affects more women (26). Localization in both OPMDs and OSCC mostly involved the tongue, floor of the mouth, and alveolar mucosa (27). In accordance with habits, tobacco could alter serum vitamin D levels through smoking by-products such as lead and cadmium (28). Moreover, tobacco use can promote alterations in the expressions of CYP24A1 and CYP27B1, which affect the enzymes that regulate circulating vitamin D levels. Furthermore, aging of the skin, a product of the free radicals in tobacco, would impact the main source of vitamin D in the body (29).

Optimal serum vitamin D levels vary according to life stage, race, ethnicity, sex, and others. No consensus was observed in the categorization of normal serum vitamin D values, but some authors agreed that vitamin D levels above 30 ng/ml are sufficient for normal function (13,14). Levels over 30 ng/ml could be necessary to maximize the effects of vitamin D on calcium, bone, and muscle metabolism. However, a range between 150 and 200 ng/ml would be associated with intoxication and adverse effects (30-32). Between 1,000 and 10,000 IU may be needed to normalize serum levels in adult patients to 30 ng/ml. However, patients with conditions such as obesity, cancer, disability, or gastrointestinal problems may require around 4,000 IU/day (29,30,33). Recommended Dietary Allowances (RDAs) for vitamin D, are 600 IU/d for 1-70 years’ persons and 800 IU/d for ages 71 year and older, corresponding to a serum 25-hydroxyvitamin D level of at least 20 ng/ml (50 nmol/liter) (34). According to Haines *et al*. (35), in patients with vitamin D deficiency, a cumulative dose of at least 600,000 IU administered over several weeks may be necessary to replenish vitamin D stores and large single doses of 300,000 to 500,000 IU should be avoided. Furthermore, the decision of otherwise healthy young adults to take vitamin D at doses of 2000 IU / day or less is unlikely to cause harm. For patients who are not at risk of developing vitamin D deficiency, sensitive sun exposure is an inexpensive way to maintain vitamin D stores.

Hypovitaminosis D increased the susceptibility of patients with OPMDs to progression to OSCC (14) and was related to an increase of adverse effects associated with OSCC treatment, increased recurrence of neoplastic lesions, and worse prognosis for patients (20). The normalization of vitamin D levels in patients with SCC increased the cytotoxic activity of natural killer cells, favoring antitumor immune response (13). In OSCC patients with given vitamin D supplementation, pro-inflammatory profiles similar to patients with OPMDs given supplementation were observed, demonstrating a relationship between tissue and plasma changes. A reduction in anti-inflammatory mediators such as adiponectin and an increase in pro-inflammatory mediators such as IL-6, IL-17, and leptin were related to low serum vitamin D levels (16). In patients with CCC, defects in the maturation of immune cells and increased levels of immature forms of myeloid suppressor cells or CD34+ progenitor cells were also associated with lower vitamin D levels. These phenomena could explain the susceptibility of tissues to develop malignant neoplasms from OPMDs (16).

Supplementation with D3 or D2 resulted in increased serum 25(OH)D levels, with a peak in the values at day 14. Increasing vitamin D levels alleviated the adverse effects associated with chemotherapy such as oral mucositis, pain, and difficulty in swallowing. This could be because vitamin D possibly stimulates the differentiation and epithelialization in the cells of the oral mucosa (14).

A VDR Tt genotype presents a significant risk for the development of OSCC, whereas a VDR tt genotype would be a protective factor. In addition, the T allele was found in all patients diagnosed with OSCC or had tumor diameters larger than 4 cm (36). The VDR Taq I genotype with the t allele has been associated with a reduced risk of developing lung cancer due to tobacco use of up to 28% (37).

The current available literature that relates hypovitaminosis D with OPMDs and OSCC is scarce, and few authors have described measurement methods used to determine vitamin D levels. Moreover, since no standardization has been implemented for vitamin D measurement both in supplemented patients and in those who do not, the risk of bias due to physiological variations is high. Despite the limitations of our study, to our knowledge, this is the first literature review establishing an association between hypovitaminosis D and OPMDs and OSCC. It is necessary to mention that due to the different types of study designs, the diversity of the methodological quality of each one and the high percentage (85%) of observational approaches, the level of evidence is still low. In addition, the publishing bias, can overestimate the real effect of vitamin D. Besides, low number of studies included (n=12) limited the extrapolation of the results. Moreover, whether genetically higher cancer risk can influence vitamin D level, namely the reverse causation, remains unknown. Further studies are needed to physiologically clarify the mechanism by which vitamin D supplements modify the immune response of patients, in addition to studies that explain the mechanism by which hypovitaminosis D increases the susceptibility to developing OSCC and how supplementation could favor the normalization of the immune system.

The findings of this review suggest that hypovitaminosis D favors the progression from OPMDs to OSCC and is associated with lower survival in patients with SCC and OSCC, greater recurrence of tumors in patients receiving surgical treatment, and increased adverse reactions associated with chemotherapy. This review has identified a trend correlating hypovitaminosis D and the expression of certain VDR genotypes, which can indicate the risk for malignant progression in patients with OPMDs due to alterations in immune response. Tobacco use, in addition to being a risk factor associated with the development of different types of cancer, was identified as a negative factor on vitamin D levels favoring the progress from OPMDs to OSCC. The use of vitamin D supplements in patients with OSCC could be used as an adjunct to primary therapy to prevent recurrence of lesions and reduce adverse reactions associated with treatment. Regarding other cancers, new evidence also sheds light on repurposing vitamin D as a potential therapeutic agent for gastric cancer prevention and treatment for example (38).
